# Real-world refractive outcomes of toric intraocular lens implantation in a United Kingdom National Health Service setting

**DOI:** 10.1186/s12886-018-0692-7

**Published:** 2018-02-06

**Authors:** Kanmin Xue, Jasleen K. Jolly, Sonia P. Mall, Shreya Haldar, Paul H. Rosen, Robert E. MacLaren

**Affiliations:** 1 Oxford Eye Hospital, Oxford Universities Hospitals NHS Foundation Trust, Oxford, UK; 2 Nuffield Laboratory of Ophthalmology, Nuffield Department of Clinical Neurosciences, University of Oxford, Level 6 West Wing, John Radcliffe Hospital, Headley Way, Oxford, OX3 9DU UK

**Keywords:** Toric intraocular lens, Toric IOL, Cataract surgery, Astigmatism

## Abstract

**Background:**

With increasing availability of toric intraocular lenses (IOL) for cataract surgery, real-world refractive outcome data is needed to aid the counselling of patients regarding lens choice. We aim to assess the outcomes of toric intraocular lens use in the non-specialist environment of a typical United Kingdom NHS cataract service.

**Methods:**

A retrospective cohort study conducted at the Oxford Eye Hospital, Oxford University Hospitals NHS Foundation Trust, UK. All patients who received a toric IOL implant over a 10 months period. Patients underwent pre-operative corneal marking, phacoemulsification and toric IOL implantation. Biometry was obtained using a Zeiss IOLMaster 500 and the toric IOLs were selected using the manufacturers’ online calculators. Post-operative refractions were obtained from optometrist’s manifest refraction or by autorefraction. The outcome measures were post-operative unaided visual acuity (UVA), spherical equivalent refraction, cylindrical correction and all complications.

**Results:**

Thirty-two eyes of 24 patients aged 21–86 years (mean 66.4, SD 14.5) were included. UVA was superior to pre-operative best-corrected visual acuity (BCVA) in 81% of eyes, same in 16% and inferior in 3%, resulting in a median improvement of 0.20 LogMAR (IQR 0.10 to 0.30). 56%, 81%, 94% and 100% of eyes were within ±0.5, ±1.0, ±1.5 and ±2.0 D of predicted spherical equivalent, respectively. Three (9%) eyes required further surgery to rectify significant IOL rotation.

**Conclusions:**

Reduced cylindrical correction and improved UVA could be expected in the majority of patients undergoing toric IOL implantation. Patients should be counselled about the risk of lens rotation.

## Background

Advances in cataract refractive surgery have enabled neutralisation of corneal astigmatism at the time of cataract surgery using toric intraocular lens (IOL) implants. Approximately 15–20% of patients presenting with cataract have > 1.5 dioptres (D) of pre-existing corneal astigmatism [1]. Before the introduction of toric IOLs (TIOL), the main method for correcting corneal astigmatism was peripheral corneal relaxing incisions (PCRI) or opposite clear corneal incisions (OCCI). However the technique is limited by variations in corneal thickness, surgical technique and scarring response between individuals, leading to unpredictable degrees of cylindrical correction and regression of refractive correction. Creating corneal incisions of accurate depths by hand, even with a guarded diamond-tipped blade, can be operator-dependent and susceptible to patient-related variables such as intraocular pressure and eye movement under local anaesthesia. Although femtosecond laser may be able to create PCRIs with a high degree of precision thereby minimising the risk of corneal perforation, PCRIs could still be associated with the risks of wound gape, corneal infection and nerve damage leading to secondary dry eye [2–4] Alternatively, astigmatic correction through excimer laser photorefractive keratectomy could be complicated by refractive error, and all the complications associated with the technique including diffuse lamellar keratitis and corneal haze [5, 6]. It is also generally not available within the UK National Health Service (NHS) and most cataract surgery centres across Europe.

The introduction of a variety of TIOLs and manufacturer validated formulae for calculating the required lens power have enabled the correction of corneal astigmatism during cataract surgery without the need for any additional incisions. Provided the eye has regular astigmatism, the rotational alignment of the TIOL with respect to the steep meridian of the cornea is critical for achieving the desired refractive outcome. Rotation of a toric IOL from its intended orientation can degrade its cylindrical corrective power by 3.3% for every 1 degree (°) off axis. Therefore, a 30° rotation of the TIOL would completely negate the effectiveness of the astigmatic correction and a misorientation of > 30° would induce additional astigmatism. Potential risk factors for misorientation of the TIOL include erroneous measurement of the pre-operative corneal astigmatism, inaccurate marking of the cornea, incomplete viscoelastic removal, IOL dialling error (e.g. due to parallax) and post-operative rotation due to wound leak or capsule contraction. Most reports on TIOL use to date, however, are from specialist cataract surgeons who are highly experienced with high volume toric lens use and their excellent results may not necessarily be representative of the wider ophthalmology community. The purpose of this study was therefore to assess the real-world outcomes of TIOL implantation in a typical public hospital performed by a mixture of surgeons as part of routine cataract surgery in the NHS.

## Methods

We conducted a retrospective analysis of all patients who received a TIOL implant at the Oxford University Hospitals NHS Foundation Trust, a tertiary referral centre in the UK, over a 10 months period. This retrospective clinical audit was conducted with local institutional review board (IRB) approval from the Clinical Effectiveness Committee, Clinical Governance and Risk Neurosciences, Orthopaedics, Trauma and Specialist Surgery (NOTSS) Division, Oxford University Hospitals NHS Foundation Trust (Datix registration no. 4916), and exempt from UK National Research Ethics Service approval (as per NHS Health Research Authority guidance). Permission was given to access patient data, which were de-identified. It adhered to the tenets of the Declaration of Helsinki. As per local policy, patients with ΔK > 2.00 dioptres (D) based on biometry obtained using a Zeiss IOLMaster 500 (Carl Zeiss AG, Jena, Germany) and consistent with previous refraction, were eligible for a TIOL. Patients in whom the previous optometry report suggested inability to fully correct visual acuity (to 6/6) with glasses underwent corneal topography using Pentacam (Oculus, Wetzlar, Germany) to rule out irregular corneal astigmatism (a contraindication to TIOL implantation in this hospital). The biometry protocol followed the recommendations within the Royal College of Ophthalmologists Cataract Surgery Guidelines 2010 (https://www.rcophth.ac.uk/standards-publications-research/clinical-guidelines). Briefly, the best of 5 repeat axial length measurements (with a minimum signal-to-noise ratio of 2.0) and the mean of 3 sets of keratometry measurements were selected for IOL power calculations. Biometry was repeated if the inter-eye axial length difference was found to be > 0.3 mm and potential causes reviewed.

**Fig. 1:**
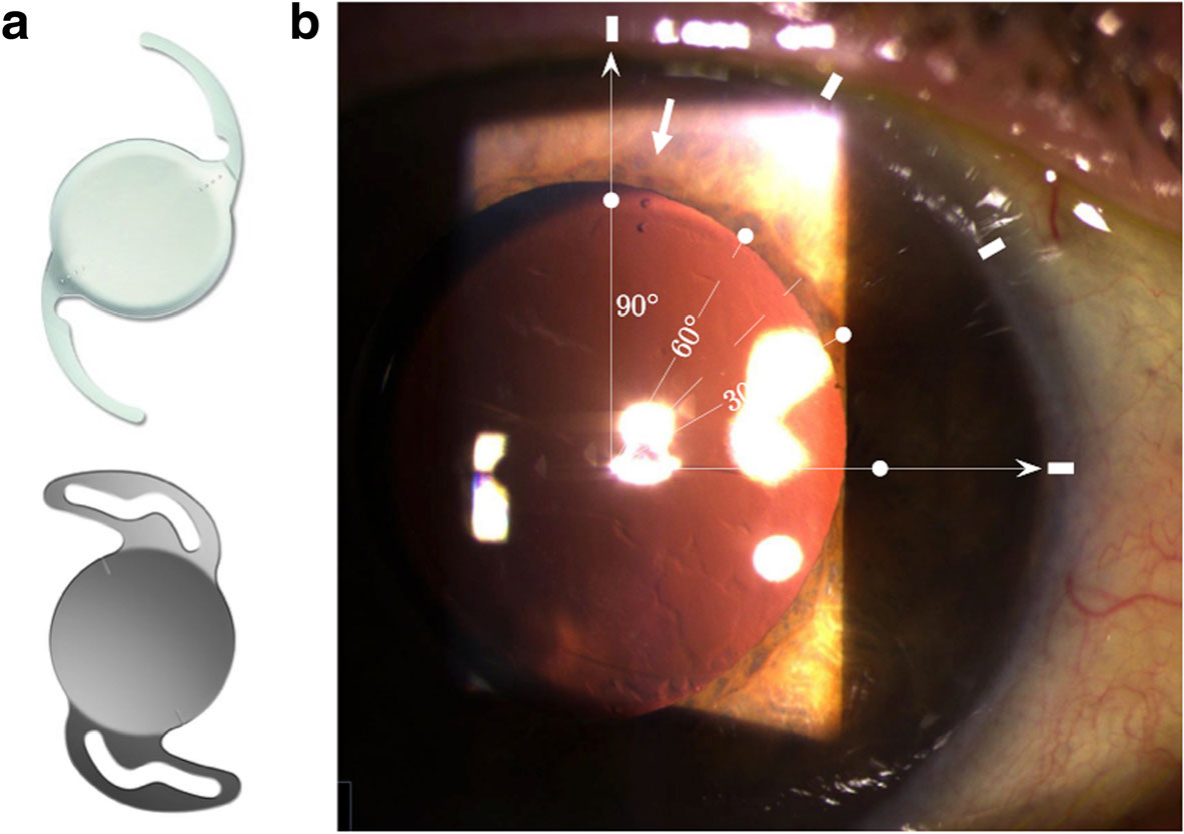
Toric intraocular lenses. **a** Schematics of the AMO Tecnis Toric Aspheric IOL with an open-loop haptic design (top) and Rayner T-flex Toric IOL with a closed-loop haptic design (bottom). **b** Post-operative slip lamp photograph of a Tecnis Toric Aspheric IOL aligned to the steep corneal meridian at 85° (arrow) with reference angle overlay

All procedures were performed by surgeons ranging from specialty registrars to consultants (attending physicians) who had experience of at least 300 cataract procedures. The standard surgical approach to cataract surgery involved micro-coaxial phacoemulsification through a 2.2 mm corneal incision. Prior to surgery, the cornea was anaesthetised and marked at 90° and 270° on the slit lamp using a sterile 20 gauge needle followed by marker pen with vertical head alignment to minimise cyclotorsion. Three types of 1-piece acrylic TIOLs were available and the choice was made based on surgeons’ preferences: Tecnis Toric Aspheric IOL (AMO, Illinois, USA), T-flex Aspheric Toric IOL (Rayner, Worthing, West Sussex, UK), or Acrysof IQ Toric IOL (Alcon, Fort Worth, USA). In one eye, a secondary Rayner Sulcoflex Toric (653 T) pseudophakic supplementary IOL was implanted (Table 1). TIOL power and orientation were chosen using the manufacturers’ online calculators, which took into account axial length (AL), keratometry values (K-values), anterior chamber depth and surgeon-induced astigmatism. Under the operating microscope, a Mendez Degree Gauge was used to mark the steep meridian of the cornea. After cataract extraction, the TIOL was injected into the capsular bag under viscoelastic (Healon, Abbott Medical Optics, Santa Ana, USA) and dialled to align with the marked steep meridian. Healon was then removed from the bag, including from behind the IOL, and the IOL alignment rechecked before the end of surgery. Post-operatively, all patients received chloramphenicol eye drops four times per day for 2 weeks and dexamethasone 0.1% eye drops four times per day for 4 weeks, and were reviewed at 2 weeks (Fig. 1).

**Table 1:** A list of 32 toric intraocular lenses (TIOL) implanted over a 10 months period

ID	Age (yr)	Eye	Co-morbidity	IOL type	IOL power (D)	Pre-op BCVA	Post-op UVA
1	67	R		Tecnis ZCT 400	20.0	0.30	0.30
2	61	R		Tecnis ZCT 225	14.0	0.30	0.00
		L		Tecnis ZCT 225	14.0	0.50	0.10
3^a^	56	R		Tecnis ZCT 400	12.0	0.30	0.00
4	76	R		Tecnis ZCT 225	18.0	0.50	0.18
5	77	L^b^		Tecnis ZCT 150	20.0	0.30	0.00
		R		Tecnis ZCT 400	19.0	0.60	0.18
6	67	R		Tecnis ZCT 400	16.5	0.30	0.00
		L	ERM	Tecnis ZCT 400	13.5	0.50	0.48
7^a^	49	R	Vitrectomised	Tecnis ZCT 300	19.5	0.50	0.30
8	67	L		Tecnis ZCT 400	16.5	0.30	0.10
		R		Tecnis ZCT 225	11.0	0.20	0.00
9	78	R		Tecnis ZCT 300	20.5	0.50	0.30
10	82	L		Tecnis ZCT 225	23.5	0.00	0.00
11	82	R		Tecnis ZCT 400	24.5	0.20	0.18
12	65	L		Tecnis ZCT 400	17.5	0.30	0.00
		R		Tecnis ZCT 400	11.0	0.30	0.18
13	41	R		Tecnis ZCT 400	26.5	2.00	0.18
		L	Amblyopia	Tecnis ZCT 400	27.5	1.00	0.18
14	60	L		Tecnis ZCT 400	26.0	0.00	0.30
		R		Tecnis ZCT 400	26.5	0.30	0.30
15	80	R	Amblyopia	Tecnis ZCT 400	27.0	0.60	0.30
16	21	R		Tecnis ZCT 300	22.5	0.48	0.00
17	78	L		Tecnis ZCT 225	31.0	0.18	0.18
18	85	L		Tecnis ZCT 400	21.0	0.30	0.18
19	82	R		Tecnis ZCT 400	17.5	0.30	0.30
20	86	L		Tecnis ZCT 300	23.5	0.30	0.10
21	53	R	Previous LASIK & PCRI	AcrySof SN60 T8	23.0	0.18	0.10
		L		AcrySof SN60 T8	22.0	0.30	0.10
22	74	L		Rayner T-flex 623	18.0	0.48	0.18
23	72	L		Rayner T-flex 623	16.0	0.30	0.18
24	74	L	Corneal scar	Rayner T-flex 623	16.5	0.30	0.18

^a^Indicate cases complicated by TIOL rotation, which were corrected surgically

^b^The left eye of patient 5 received a low powered toric IOL (Tecnis ZCT 150) to correct a ΔK of 1.41 D (which is lower than the ΔK > 2.00 D inclusion criteria). This was at the clinician’s discretion as the patient had already received a higher powered toric IOL in the fellow eye

Outcome measures were pre- and post-operative visual acuities (unaided and best-corrected), refractions (obtained either by manifest refraction by an optometrist or by autorefraction), and all complications. The minimum follow-up of post-implantation refractive outcomes was 2 weeks. The predicted spherical equivalent (SE) following toric IOL implantation was compared with the post-operative refraction. Statistical analyses were performed using Microsoft Excel (Microsoft Corporation, Washington, USA), StatsDirect (StatsDirect Ltd., UK), and GraphPad Prism 7.0a (GraphPad Software Inc., California, USA). Paired t-tests were used when normality was noted, otherwise the Wilcoxon signed rank test was used for statistical comparisons. The sphero-cylindrical refractions were converted to vector notation and analysed using modified Alpins and Goggin’s methodology for vector analysis [7]. For the vector analysis, the cylindrical power was resolved into its *X* and *Y* components using *sine* and *cosine* trigonometric functions. The difference in power from predicted post-operative refraction was calculated for the spherical equivalent, astigmatic refraction in both *X* and *Y* components. The surgically induced refractive correction (SIRC) was calculated. Together these allowed calculation of total error magnitude (TEM) to reflect the error in power of the astigmatism, and angle error (orientation). Pre- and post-operative blurring strength was also calculated to evaluate the effect of the error on subjective vision [8].

## Results

Thirty-two eyes of 24 patients with a mean age of 66.4 years (SD 14.5 yr., range 21–86 yr) were included in the study: 27 eyes received a Tecnis Toric Aspheric IOL, 3 eyes received a Rayner T-flex Aspheric Toric IOL, and 2 eyes received an Acrysof IQ Toric IOL (Table 1). Phacoemulsification and toric IOL implantations were performed by 15 different surgeons (3 consultants, 2 associate specialists, 4 fellows and 6 specialty registrars). One patient received a secondary Rayner Sulcoflex toric pseudophakic IOL to correct residual refractive error (− 2.75/− 0.75 × 1°) following cataract surgery, achieving a final refraction of 0.00/− 0.75 × 10° with an UVA of 0.00 LogMAR. This case was however excluded from the cohort analyses as the ‘piggy-back’ sulcus TIOL was considered not to be comparable with other aforementioned ‘in-the-bag’ TIOLs.

There were no general cataract surgery related complications, such as posterior capsule rupture, zonular dialysis or endophthalmitis. However, significant TIOL rotation was found in three (9%) eyes post-operatively, which required IOL repositioning. All three eyes received a Tecnis Toric IOL. In the first case, the TIOL was found to have rotated by 10° 1 week post-operatively. IOL repositioning was performed with final unaided visual acuity (UVA) of 0.60 LogMAR and BCVA of 0.00 LogMAR, consistent with a refractive target of − 2.44 D. The second case was in a vitrectomised eye following previous retinal detachment repair. The toric IOL rotated by 30° and was repositioned at 2 weeks. The IOL orientation remained stable at 3 months follow-up, achieving a final UVA of 0.30 LogMAR, consistent with a refractive target of − 2.12 D. The third case was also an eye that had undergone vitrectomy for retinal detachment repair. The TIOL was found to have rotated by 25° immediately after surgery, which recurred despite IOL repositioning twice in quick succession (over 2 h). The TIOL was eventually exchanged for a single focus IOL combined with PCRIs, leading to an UVA of 0.10 LogMAR. As the TIOL was ultimately removed in this case, it was included in the complication analysis but excluded from refractive outcomes analysis. Vector analysis was conducted using the final refractions following TIOL repositioning.

In the 32 eyes that received TIOLs, 26 (81%) eyes achieved an improvement in post-operative UVA compared with the pre-operative best-corrected VA (BCVA), 5 (16%) eyes showed no change, and 1 (3%) eye showed a reduction. The median improvement in post-operative UVA over pre-operative BCVA was 0.20 LogMAR (IQR 0.10 to 0.30), equivalent to 2 Snellen lines. The mean post-operative LogMAR UVA ranged from 0.00 to 0.48. In the one eye that showed a poorer post-op UVA than pre-op BCVA, 2.00 D of residual cylinder was found and the UVA (0.30 LogMAR) improved to 0.20 with pinhole.

**Fig. 2:**
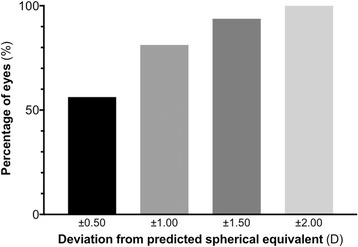
Frequency distribution showing the percentage of eyes achieving different levels of deviation of the post-operative spherical equivalent (SE) from the target SE following toric IOL implantation

**Fig. 3:**
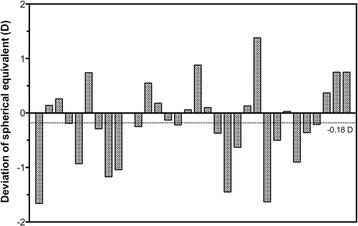
Bar chart showing the post-operative deviation of spherical equivalent (SE) from the predicted SE for each eye (*n* = 32). Positive values indicate over-correction while negative values indicate under-correction of SE. The median deviation was − 0.18 D (dotted line)

In terms of final refractive outcome, 18 eyes (56%) reached within ±0.50 D of the predicted SE, 26 eyes (81%) reached within ±1.00 D, 30 eyes (94%) reached within ±1.50 D, and 32 eyes (100%) reached within ±2.00 D (Fig. 2). The range of deviation of the post-operative SE from the predicted SE (based on manufacturers’ IOL calculators) was − 1.66 to + 1.38 D with a median of − 0.37 D (Fig. 3).

**Fig. 4:**
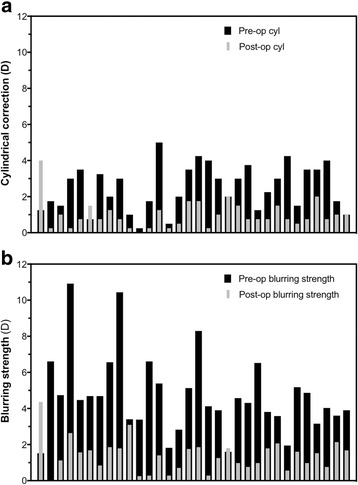
Refractive correction following toric IOL implantation. **a** Bar chart showing the difference between pre- and post-operative cylindrical corrections in each eye (n = 32). **b** Bar chart showing changes in subjective blur experienced by each eye (n = 32) pre- and post-TIOL implantation

The pre-operative median ΔK was 2.94 D (interquartile range 2.41 to 3.24). The pre-operative median refractive cylinder was 2.63 DC (IQR 1.50 to 3.50) whilst the post-operative median refractive cylinder was 0.75 DC (IQR 0.50 to 1.44), representing a statistically significant reduction (Wilcoxon signed rank test: *p* < 0.0001) (Fig. 4a). Twenty-seven (84%) eyes obtained a reduction in refractive cylindrical correction. However, two (6%) eyes showed no change in refractive cylinder, whilst three (9%) eyes showed an increase in refractive cylinder: from − 1.25 to − 4.00 DC, − 0.75 to − 1.50 DC, and − 1.00 to − 2.25 DC, respectively. The first of these three eyes obtained the same post-operative UVA as pre-operative BCVA (0.50 LogMAR), and was not associated with significant IOL rotation - measured to be 6°. The other two eyes both demonstrated improvement of post-op UVA compared with pre-op BCVA despite the increases in refractive cylinder, which was likely due to removal of lens opacity from cataract extraction.

Blurring strength was calculated as the geometrical representation of the sphero-cylindrical refractive errors and represented the subjective blur experienced by the patient as a result of their refractive error. A significant reduction in median blurring strength from 4.39 D (IQR 3.49 to 5.28) pre-operatively to 1.46 D (IQR 0.76 to 1.80) post-operatively was seen in this cohort of eyes following TIOL implantation (95% CI for change in blurring strength = 2.55 to 3.99 D, *p* < 0.0001) (Fig. 4b).

**Fig. 5:**
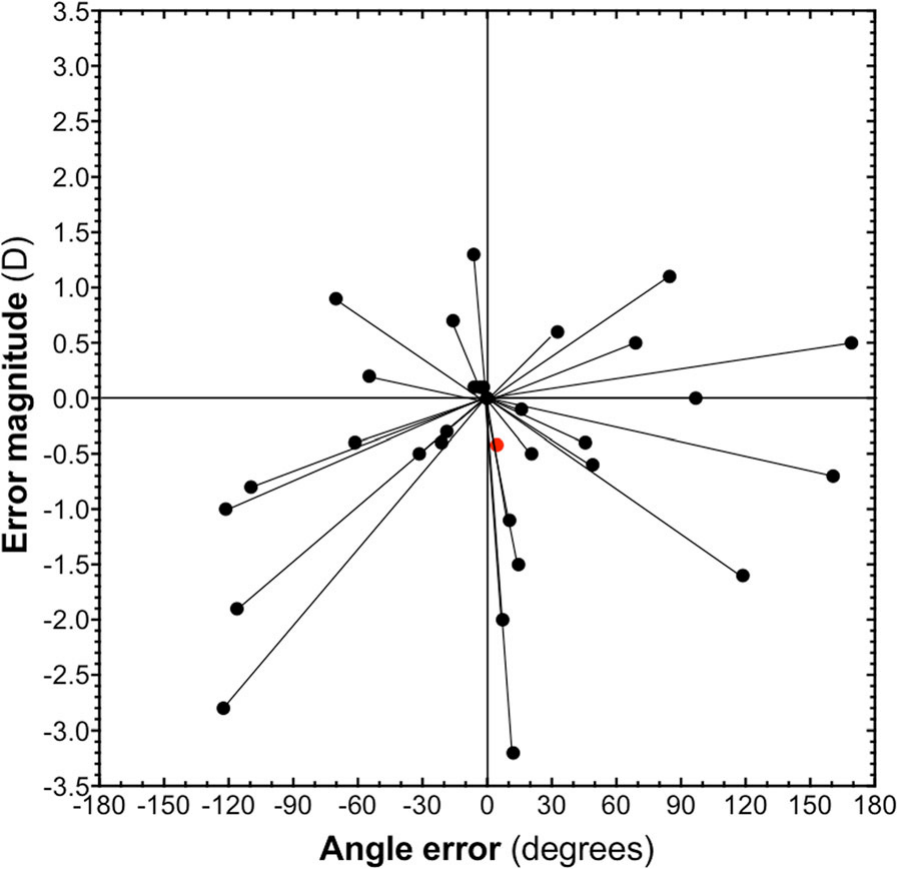
Vector analysis showing the magnitude and angle of the error in astigmatic correction of individual eyes (black dots). The combined result is an error vector with magnitude of − 0.42 D and angle of 4.63° (red dot), indicating an overall tendency towards under-correction of astigmatism

The discrepancy between achieved astigmatism and pre-operative astigmatism for each individual could be expressed as an error vector and subjected to vector analysis in which polar coordinates (of cylinder power and axis) were converted to Cartesian values (Fig. 5) [7]. The combined error vector of the cohort had a magnitude of − 0.42 D and angle of 4.63°, indicating a tendency for slight under-correction of astigmatism.

## Discussion

This study has shown that TIOLs could provide predictable correction of the sphero-cylindrical error in cases of low to moderate corneal astigmatism. It represents one of the largest cohorts assessing the refractive outcomes and rotational stability of toric IOLs within a real-world mixed surgeon public health service (NHS) setting.

The post-operative unaided distance visual acuity and refractive cylindrical corrections of this cohort were comparable to other published studies of 1-piece acrylic toric IOLs (Table 2). For instance, Hirnschall et al. [9] evaluated the outcomes of 30 eyes that received the Tecnis Toric IOL and found a post-op UVA of − 0.05 logMAR at 3 months and mean IOL rotation of 2.7° (SD 3.0). Tsinpoulos et al. [10] demonstrated that 90% of a cohort of 29 eyes that received the Alcon Acrysof toric IOL achieved UVA ≥0.30 logMAR and mean post-operative astigmatism of 0.64 D (SD 0.61, range 0 to 2.5). Entabi et al. [11] showed that 33 eyes that received the Rayner T-flex toric IOL achieved mean post-operative UVA of 0.28 logMAR (SD 0.23) and mean astigmatism of 0.95 (SD 0.66). They also found a mean difference between targeted and actual IOL cylinder axis of 3.44° (range 0 to 12). The apparent increase (*n* = 3) or lack of reduction (*n* = 2) in refractive cylinder observed in some individuals in this study were most likely the result of large degrees of corneal astigmatism, which were previously neutralised by the lens astigmatism, but were unmasked by cataract extraction and only partially neutralised by the TIOL (Fig. 4). The specific reasons for this may include (i) selection of a slightly underpowered TIOL, (iii) small degrees of TIOL rotation or decentration (which can be difficult to detect), (iv) surgically-induced astigmatism, or (iv) uncorrected posterior corneal astigmatism or corneal ectasia. Despite apparently similar overall post-operative refractive cylindrical corrections obtained by this and other studies, the differences in UVA achieved may be partly related to differences in patient age, ocular co-morbidities (e.g. two eyes had mild amblyopia, 1 eye had ERM and 1 eye had a corneal scar in this cohort – Table 1), and differences in the stringency by which eyes with irregular corneal astigmatism were excluded. Only three patients in this study underwent Pentacam corneal topography to definitively rule out irregular astigmatism, but since then it has been incorporated as our standard protocol for patients undergoing toric IOL implantation in order to optimise refractive outcomes.

**Table 2:** Comparison of toric IOL implantation studies

Study	Eyes	Age (years ±SD or range)	Toric IOL	Post-op UVA (logMAR) ± SD	Pre-op cyl (D) ± SD	Post-op cyl (D) ± SD	Max IOL rotation (°)
This study	32	66.6 ± 14.5	Tecnis Toric (*n* = 27), Rayner T-flex (n = 2), Alcon Acrysof toric (n = 3)	0.16 ± 0.12	2.45 ± 1.2	1.04 ± 0.79	30
Tsinpoulos et al. [10]	29	63 ± 5.4	Alcon Acrysof toric	≥0.30 (90%)	2.38 ± 0.91	0.64 ± 0.61	8.4
				≥0.10 (66%)			
Hirnschall et al [9]	30	67 (36–85)	Tecnis Toric	−0.05	1.80 ± 0.50	0.90 ± 0.40	13.7
Entabi et al. [11]	33	81 ± 8.9	Rayner T-flex	0.28 ± 0.23	3.35 ± 1.20	0.95 ± 0.66	17

TIOL rotation following implantation is a well-known phenomenon and modern lens designs have sought to reduce this risk [12]. The Tecnis Toric and Alcon Acrysof IQ TIOLs used in this study both have an open-loop haptic design, whereas the Rayner T-flex TIOL has a closed-loop haptic design. However, there does not appear to be any strong linkage between these two types of haptic design and rotational stability of the IOLs [9, 11, 13]. Long-term stabilisation of the toric IOL is thought to result of fusion between the anterior and posterior capsules, which trap it in a permanent orientation, however factors that could influence this fusion include capsulorhexis size, residual viscoelastic in the bag, IOL design and material [13]. Older TIOLs were often made of silicone and were associated with poor capsular adhesion and high post-operative misalignment rates, whereas the modern TIOLs (as used in this study) are made of acrylic, which appears to induce stronger capsular adhesions [14]. The three cases of significant TIOL rotation encountered in this cohort appear to indicate previous vitrectomy as a risk factor. One possible explanation is that post-vitrectomy eyes may be associated with zonular weakness, e.g. secondary to zonular stretching by expansile gas, zonular stress from indentation, or zonular damage during vitreous base shaving [15]. If a sector of zonular weakness lies close to the desired TIOL meridian, the resulting uneven tension around the capsular bag could provide the torque for IOL rotation. It may explain why IOL misalignment recurred almost immediately after IOL repositioning in one of our cases. This raises the question whether previous vitrectomy might be a relative contraindication to TIOL implantation but a capsule tension ring may be used to prevent TIOL rotation secondary to zonular weakness in those eyes. Interestingly, a recent close observational study of 72 eyes following Tecnis Toric IOL implantation revealed that most (around 60%) of the IOL rotation occurred within 1 h after surgery and further rotation was minimal after 1 week [16]. While TIOL rotation may be detected early, it remains unclear whether immediate repositioning would carry a significant risk of recurrence whereas allowing some capsule-IOL adhesion to develop over a short time period may help to stop the IOL from rotating again.

Vector analysis of the difference between calculated and actual refractive outcomes showed a combined error vector of − 0.42 D in magnitude with an angle of 4.63°, which would suggest a tendency towards under-correction of astigmatism and slight clockwise rotation of the IOL. There could be a range of explanations for the under-correction of astigmatism. For instance, small IOL misalignments could result from inaccurate corneal markings, ocular cyclotorsion on the slit lamp, or decentration of the IOL with regard to angle kappa. Other potential sources of error in toric IOL calculations could arise from incorrect calculation of the cylinder power using standard biometry formulae in cases where the effective lens position or the relationship between anterior and posterior corneal curvature was not as predicted. Newer techniques have now emerged to ensure accurate intraoperative alignment of toric IOLs, such as iris fingerprinting in which high-resolution iris photograph obtained pre-operatively can assist with corneal marking, and intra-operative wavefront aberrometry (IWA).

In summary, the use of the toric IOLs within routine NHS setting consistently improved UVA and reduced blurring strength in patients with significant corneal astigmatism, thereby facilitating spectacle-independence. The median refractive cylinder was reduced from 2.63 D pre-operatively to 0.75 D post-operatively. Three out of 32 (9%) eyes required IOL repositioning to correct lens rotation with previous vitrectomy being a common risk factor in two-thirds of the cases. Cost-benefit analysis of the widespread use of TIOL within a public healthcare setting would need to balance the quality of life improvements associated with spectacle-free vision with the additional costs associated with pre-operative corneal topography and toric IOL implantation, as well as the potential costs associated with lens repositioning/explantation in a proportion of cases.

## Conclusions

The post-operative unaided visual acuities (UVA) and refractive outcomes of toric intraocular lens implantation were analysed in a high-volume public health service setting. Three different toric IOLs were implanted: Tecnis Toric, Alcon Acrysof Toric and Rayner T-flex. Post-operative UVA was superior to pre-operative best-corrected VA in 26 of 32 (81%) eyes. Significant IOL rotation occurred in 9% of cases with previous vitrectomy being a common risk factor in two thirds of the cases.

## Abbreviations

BCVA: Best Corrected Visual Acuity
IOL: Intraocular lenses
NHS: National Health Service
OCCI: Opposite clear corneal incisions
PCRI: Peripheral corneal relaxing incisions
TIOL: Toric IOLs
UVA: Unaided visual acuity
